# The *Arabidopsis* CYSTM α 5′ UTR Increases Protein Production from Transgenes in Plants and Bacteria

**DOI:** 10.3390/genes17050520

**Published:** 2026-04-28

**Authors:** Jasjyot Singh Khanduja, Xingyu Wu, Jun Li, Iain R. Searle

**Affiliations:** School of Biological Sciences, Adelaide University, Adelaide, SA 5005, Australia; jasjyotsingh.khanduja@adelaide.edu.au (J.S.K.);

**Keywords:** post-transcriptional regulation, mRNA translation, *Arabidopsis thaliana CYSTM1* (AT1G05340), 5′ untranslated region (5′UTR)

## Abstract

**Background**: Translational regulation constitutes a critical layer of gene expression control in plants, yet the contribution of endogenous 5′ untranslated regions (5′ UTRs) to translational efficiency remains incompletely defined. While viral and synthetic leader sequences have been widely used to enhance protein production, comparatively few native plant 5′ UTRs have been systematically characterised. The objective of this study was to identify and functionally evaluate endogenous plant 5′ UTR elements that promote translation through post-transcriptional mechanisms. **Methods**: A 79-nucleotide fragment (*CYSTM α*) derived from the 5′ UTR of *Arabidopsis thaliana CYSTM1* (AT1G05340) was cloned upstream of reporter genes and assessed using dual-luciferase assays in transient expression systems (*Nicotiana benthamiana* and *A. thaliana*) and in stable transgenic *Arabidopsis* lines. Translational activity was further evaluated in monocot wheat germ extract and in *Escherichia coli*. Transcript abundance was quantified by qRT-PCR. Publicly available ribosome profiling and m^6^A datasets were analysed to assess translational efficiency and RNA modification status. **Results**: In *N. benthamiana* and *A. thaliana*, *CYSTM α* increases reporter protein production 3–7 fold relative to the control and 30–130% above the benchmark Tobacco Mosaic Virus (TMV) Ω leader, without altering mRNA abundance. The *CYSTM α* sequence also enhances luciferase translation in monocot wheat germ extract and elevates translation 5-fold in *E. coli*. *CYSTM α* contains three motifs that may promote translation, namely three CAA repeats that are associated with translation initiation, an AMAYAA motif that is associated with eIF3 binding, and two N6-adenosine DRACH sites that are associated with cap-independent translation. Additionally, ribosome profiling revealed high translational efficiency (TE = 3.25) of native *CYSTM1*. **Conclusions**: *CYSTM α* represents a compact endogenous 5′ UTR element that enhances translation across multiple experimental systems. These findings expand the repertoire of plant-derived translational enhancers and provide insight into sequence features associated with efficient mRNA translation in plants.

## 1. Introduction

Gene expression in plants is regulated through multilayered genetic mechanisms, with post-transcriptional control emerging as a major determinant of variation in protein output [1,2]. Among these regulatory layers, 5′ untranslated regions (5′ UTRs) have central roles in modulating ribosome recruitment, initiation efficiency, RNA structure, and interactions with RNA-binding proteins or RNA modifications [1,3,4]. Despite their significance, few endogenous plant 5′ UTRs have been systematically examined for their contributions to translational regulation, and the genetic features that define highly active plant leaders remain poorly characterised.

Transgenes provide a tractable genetic framework for dissecting these regulatory principles, as their expression can be precisely manipulated through defined cis-regulatory elements. Transgene expression is regulated by different elements, including promoters, 5′ and 3′ untranslated regions (UTRs), introns, and terminators [2,5,6,7]. These elements modulate transcriptional activity, mRNA stability, and translational efficiency [8,9]. In addition to these cis-regulatory elements, endogenous processes such as RNA silencing, histone modifications, and co-transcriptional RNA processing also play important roles in regulating transgene performance. It is well established that transgene components such as promoters, 5′ and 3′ untranslated regions (UTRs), introns, and terminators play crucial roles in regulating transcription efficiency, RNA stability, and translation rates [8,9,10,11,12]. In addition, other cellular processes such as RNA silencing, histone modifications and co-transcriptional RNA modifications can also impact transgene expression [4,13,14,15,16,17]. Notable examples of translational enhancers used in plant transgenes include the Tobacco Mosaic Virus (TMV) omega (Ω) sequence, *AtADH* 5′UTR, synJ, *NtADH* 5′ UTR, and *OsADH* 5′ UTR [18,19,20,21,22,23,24].

Here we identify a short 79 nt sequence derived from the 5′ UTR of the *A. thaliana CYSTM1* (Cysteine-rich Transmembrane module) gene (AT1G05340) that functions as a potent translational enhancer. CYSTM proteins constitute a conserved family associated with stress tolerance and membrane function in plants, although their regulatory features at the RNA level remain poorly characterised [25]. This sequence significantly boosts transgene expression across multiple systems, including in planta (both in vivo and in vitro) and in bacteria (*E. coli*), without altering mRNA abundance. Comparative analyses reveal that the *CYSTM1* 5′ UTR enhancer consistently outperforms the widely used TMV Ω enhancer, achieving up to a two-fold increase in transgene expression.

The identification of this compact and broadly active regulatory element expands our understanding of plant translational control and provides a new tool for dissecting the genetic basis of mRNA translation in plants.

## 2. Materials and Methods

### 2.1. Plant Materials

*A. thaliana* (Columbia ecotype) plants were cultivated in controlled environment rooms (Phoenix Biosystems, Edwardstown, SA, Australia), where the temperature was maintained at 21 °C under Heliospectra ELIXIA LED lights (Gothenburg, Sweden)with all 4 tunable LED channels—450 nm, 660 nm, 735 nm and 5700 K—set to 200 µmol m^−2^ s^−1^. Plants were grown under long-day photoperiod conditions of 16 h light and 8 h darkness for 3 weeks. *N. benthamiana* (RDR6i) plants were germinated on soil in a master pot and then transferred to individual pots after 10 days. Plants were grown in a Phoenix Biosystem controlled environment room with metal halide lights (100 µmol/m^2^/s) for 3 weeks.

### 2.2. Plasmid Construction

Double-stranded fragments containing either the full-length 110 nt 5′ UTR *CYSTM1* or a short 79 nt CYSTM α sequence flanked by SalI and PstI restriction sequences were synthesised as gBlocks by Integrated DNA Technologies (IDT), which were double-digested using SalI-HF (NEB) and PstI-HF (NEB) restriction enzymes before immediately cloning 5′ of the Firefly luciferase initiation codon and 3′ of the CaMV 35S promoter of the dual-luciferase plasmid 35S:*LUC* (pGrDL-SPb, Addgene #83205). In p35S:RUBY (Addgene #160908), the 79 nt CYSTM α sequence was immediately cloned 5′ of the initiating *CYP76AD1* ORF [26].

### 2.3. Transient Expression by Agroinfiltration in N. benthamiana Leaves

Transient infiltration was performed using the modified protocol [27]. Briefly, three mature leaves from 3 to 4-week-old *N. benthamiana* plants were selected for agroinfiltration. To ensure even distribution of the bacterial suspension (1X MS salt (4.3 g/L); 10 mM MES; 3% sucrose; 200 uM acetosyringone, pH = 5.6), infiltration was performed over the entire leaf area via the abaxial surface using a 1 mL plastic syringe without a needle. Following infiltration, the plants were incubated in a controlled growth environment for two days prior to downstream experiments.

### 2.4. Transient Expression by Agroinfiltration in A. thaliana Leaves

*Arabidopsis* transient expression by agroinfiltration was performed according to a slightly modified protocol described in [28]. The protocol was followed exactly up to the infiltration step; however, an extended dark period of 60 h instead of 24 h was applied to the plants.

### 2.5. Dual-Luciferase Assays

Quantitative dual-luciferase assay was performed as per the protocol described in [27]. Qualitative imaging of *N. benthamiana* leaves to detect luciferase activity was performed as described in [29] using the Promega Dual-Luciferase^®^ bioluminescence reporter system (Madison, WI, USA, Cat No. E1910).

### 2.6. Dual-Luciferase Assay for E. coli Cells

*E. coli* cells containing either the 35S:*LUC*, 5′ TMV Ω, or 5′ *CYSTM α* vectors were streaked onto LB plates from a −70 °C glycerol stock; single colonies were transferred to LB broths and grown for approximately 12 h at 37 °C. The cultures were diluted to an OD600 of 0.2 and grown until the OD600 was 0.6. An amount of 1.5 mL of bacterial culture was pelleted at 14,000× *g* for 3 min. The supernatant was discarded, and the pellet was resuspended in 50 µL of passive lysis solution provided in the Promega Dual-Luciferase^®^ bioluminescence reporter system (Cat No. E1910). An amount of 15 µL of this solution was used to perform the quantitative dual-luciferase assays in a Promega GloMax^®^ Discover (Madison, WI, USA), according to the manufacturer’s protocol.

### 2.7. Betalain (RUBY) Extraction and Quantification

Spectrophotometric quantification of betacyanins produced by RUBY in plant tissue was performed as per the protocol described in [30].

### 2.8. RNA Extraction, cDNA Synthesis, and Quantitative PCR

Total RNA was extracted from 5 mg discs of *N. benthamiana* leaf tissue using the Spectrum Plant total RNA kit (Sigma-Aldrich, St. Louis, MO, USA), and contaminating DNA was removed using DNase (Sigma-Aldrich). cDNA synthesis for qRT-PCR was performed using oligo dT20 primer and an Invitrogen SuperScript IV kit (Invitrogen, Carlsbad, CA, USA) as per the manufacturer’s instructions. PCR amplification was performed on a QuantStudio^TM^ 7 Flex Real-Time PCR System (Applied Biosystems^TM^, Waltham, MA, USA). The oligonucleotides used in this study were F-Luc_F: 5′ TGCACATATCGAGGTGGACATC 3′, F-Luc_R: 5′ TGCCAACCGAACGGACAT 3′, R-Luc_F: 5′ CTGGCTCAATATGTGGCACAA 3′, and R-Luc_R: 5′ CATGGTAACGCGGCCTCTT 3′. Amplification values were calculated using the ∆∆CT method.

### 2.9. Protein Analysis by Western Blotting

Proteins were separated on two SDS-PAGE gels, with one gel Coomassie-stained as a loading control and the other transferred to PVDF membranes (Mini Trans-Blot Cell, Bio-Rad, Hercules, CA, USA) overnight at 4 °C using standard transfer buffer (48 mM Tris, 39 mM glycine, 20% methanol, 1.3 mM SDS, pH 9.2). Membranes were probed with anti-luciferase antibody (Sigma-Aldrich, L0159) and HRP-conjugated rabbit secondary antibody (Abcam, ab6721) (Hangzhou, China). Signals were detected with SuperSignal™ West Pico PLUS (Thermo Fisher, Waltham, MA, USA, 34580) and imaged on a ChemiDoc XRS+ (Bio-Rad). Band intensities were quantified with ImageJ (Version 2.17.0) (NIH) and analysed using GraphPad Prism (version 10).

### 2.10. Statistical Analysis

All experiments were performed with at least three independent biological replicates. Data are presented as mean ± standard deviation (SD). Statistical significance was assessed using one-way analysis of variance (ANOVA) followed by Tukey’s multiple comparison test to compare all groups. For pairwise comparisons, a two-tailed Student’s *t*-test was used where appropriate. Statistical analyses were performed using GraphPad Prism (version 10). Differences were considered statistically significant at *p* < 0.05.

## 3. Results

### 3.1. Identification of a Translation Enhancer Sequence in the 5′ UTR of CYSTM by Transient Expression in N. benthamiana Leaves

**Figure 1:**
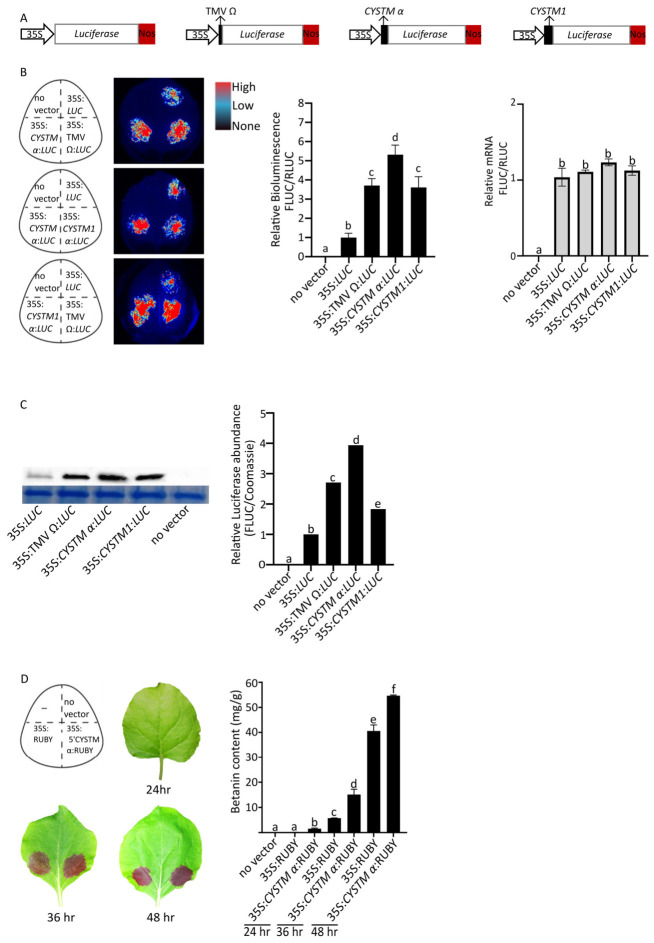
*CYSTM1* enhances dual-luciferase and RUBY transgene expression in *N. benthamiana* leaves. (**A**) Schematic representation of constructs used: 35S:*LUC* (control), 35S:TMV Ω:*LUC* (67 nt), 35S:*CYSTM α:LUC* (79 nt), and 35S:*CYSTM1*:*LUC* (110 nt). The CaMV 35S promoter is abbreviated to 35S. (**B**) (**Left**) Schematic of agroinfiltration sites and representative false-coloured bioluminescence images of leaves at 72 h post-infiltration. Imaging was performed using a CCD camera (Bio-Rad) under high-sensitivity conditions. Exposure time was adjusted for each leaf as required. In the false-colour scale, red indicates high bioluminescence, blue indicates low bioluminescence, and black indicates no detectable bioluminescence. (**Middle**) Dual-luciferase assay quantification of reporter activity. Data represent mean ± SEM (*n* = 5 biological replicates; experiment repeated three times). Statistical significance was determined by one-way ANOVA (*p* < 0.01), with different letters (a–d) indicating significant differences. (**Right**) qRT-PCR analysis of Firefly luciferase transcript levels. (**C**) (**Left**) Western blot analysis of luciferase protein accumulation. (**Right**) Densitometric quantification of band intensity using FIJI. Statistical significance is indicated by letters (a–e). (**D**) Temporal quantification of betanin accumulation in leaves expressing 35S:RUBY (control) or 35S:5′ CYSTM α constructs at 24, 36 and 48 h post-infiltration. Data represent mean ± SEM. Statistical significance was assessed by one-way ANOVA (*p* < 0.01), with different letters (a–f) indicating significant differences.

Previously, our lab mapped RNA modification sites on mRNA purified from *A. thaliana* seedlings [31]. To test whether any of these modifications could impact translation, 11 DNA fragments encoding the modified RNA regions were cloned into a Firefly luciferase (F-Luc) reporter either upstream (5′ UTR) or downstream (3′ UTR) of the F-Luc coding sequence. These constructs were agroinfiltrated into *N. benthamiana* leaves and assayed for bioluminescence (Table A1). While 10 showed bioluminescence similar to the control, one fragment, 5′ *CYSTM1 α*/AT1G05340, increased bioluminescence by 5-fold (Figure 1B). We further investigated this enhanced bioluminescence as well as an established viral enhancer.

To compare the 79 nt 5′ *CYSTM α* enhancer with its native context and with a well- established viral enhancer, we cloned the full 110 nt *CYSTM1* 5′ UTR (35S*:CYSTM1*:*LUC*) and the 67 nt TMV Ω enhancer (35S:TMV Ω:*LUC)* upstream of the *Photinus pyralis* (firefly) luciferase gene and used the *Renilla reniformis* (sea pansy) luciferase as an internal control (Figure A2). After Agrobacterium-mediated transient expression in *N. benthamiana* leaves, we observed a 4-fold higher firefly bioluminescence from both the TMV Ω enhancer (35S:TMV Ω:*LUC*) and *CYSTM1* 5′ UTR (35S*:CYSTM1*:*LUC*) compared to the no-insert 35S:*LUC* (Figure 1B). In comparison, after agroinfiltrating the 79 nt *CYSTM1* 5′ UTR enhancer (35S*:CYSTM α*:*LUC*), we observed a 5.5-fold increase in bioluminescence compared to the control (Figure 1B). The short 79 nt sequence gave 30% more firefly bioluminescence than the full-length *CYSTM1* 5′ UTR (Figure 1B).

Next, we conducted qRT-PCR to quantify the Firefly luciferase steady-state mRNA abundance and observed no difference in the mRNA abundances between 35S:*LUC*, *35S:CYSTM α*:*LUC*, 35S:TMV Ω:*LUC*, or 35S:*CYSTM1*:*LUC* (Figure 1B). In contrast, western blot and ImageJ analysis revealed approximately four-fold higher LUCIFERASE protein from the 35S:*CYSTM α*:*LUC* transgene compared to the control, while 35S:TMV Ω:*LUC* and 35S:*CYSTM1*:*LUC* showed 2.5 and 1.8 fold increases in LUCIFERASE protein, respectively (Figure 1C). We then calculated the translation efficiency (TE) as the ratio of normalised protein abundance to mRNA abundance, which was elevated by approximately 4.7-fold relative to the control. Together, these results suggest that 35S*:CYSTM α*:*LUC* increases protein translation.

Next, we tested if the 5′ *CYSTM α* sequence enhanced expression of a complex polycistronic expression transgene, named RUBY [26]. RUBY consists of three betalain biosynthetic genes, CYP76AD1, DODA, and a Glucosyltransferase, linked by two 2A peptides that are driven by a single promoter [26]. We fused the 5′ *CYSTM α* sequence upstream of the CYP76AD1 coding sequence and transiently expressed the new transgene and the control vector in the leaves of *N. benthamiana*. At the 36 and 48 h time points, we observed an approximately 1.5-fold increase in the leaf betalain content, demonstrating that the 5′ *CYSTM α* sequence enhances transgene expression from a complex polycistronic transgene (Figure 1D).

### 3.2. 5′ CYSTM α Sequence Enhances Translation of Luciferase in A. thaliana and TnT^®^ Coupled Wheat Germ Extract System

Transient expression in *A. thaliana* leaves is often challenging due to the variable and low efficiency [32]. Following a slightly modified protocol described in [28], we obtained consistent transient expression under our growth conditions. To quantify transgene expression, we used the Dual-Luciferase^®^ Reporter Assay. We observed a 3-fold increase in Firefly luciferase activity from both of the 35S:*CYSTM α*:*LUC* and 35S:*CYSTM1*:*LUC* constructs compared to the control, and a 2.2-fold increase compared to 35S:TMV Ω:*LUC*.

**Figure 2:**
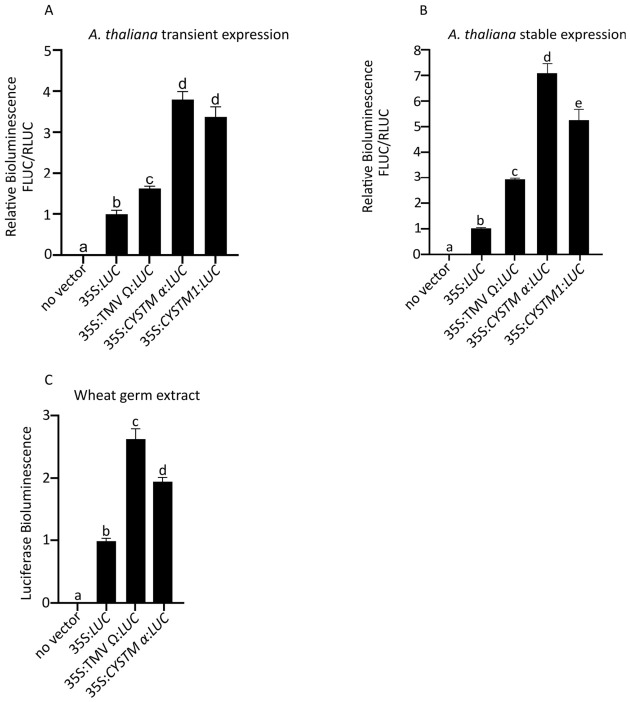
*CYSTM1* enhances dual-luciferase expression in *A. thaliana* and the TnT^®^ Coupled Wheat Germ Extract System. (**A**) Quantitative dual-luciferase assay results are shown for leaves expressing 35S:*CYSTM α*:*LUC* compared to the control constructs 35S:*LUC* and 35S:TMV Ω:*LUC*. The experiment included five biological replicates and was repeated at least three times. Error bars represent the standard error of the mean. Statistical significance is denoted by letters (a–d, *p* < 0.01, one-way ANOVA). (**B**) Quantitative dual-luciferase assay results from stably transformed T_1_ *A. thaliana* lines expressing different constructs. The experiment included five biological replicates. Error bars indicate the standard error of the mean. Statistical significance is indicated by letters (a–e) (*p* < 0.01, one-way ANOVA). (**C**) Luciferase activity in an in vitro TnT^®^ Coupled Wheat Germ Extract System for the 35S:*LUC*(control), 35S:TMV Ω:*LUC*, and 35S:*CYSTM α*:*LUC*. Error bars represent the standard error of the mean. Statistical significance is indicated by letters (a–d) and was determined using one-way ANOVA (*p* < 0.01).

Additionally, in stable transgenic *Arabidopsis* plants, we detected a 7-fold increase in the FLUC/RLUC ratio from 35S:*CYSTM α*:*LUC* relative to the control, and a 2.3-fold increase compared to the 35S:TMV Ω:*LUC* (Figure 2B). Notably, 35S:*CYSTM1*:*LUC* produced 60% higher bioluminescence than the 35S:TMV Ω:*LUC* but was 30% lower than the *CYSTM α*. Since the enhancer effect of the full-length *CYSTM* 5′ UTR (35S:*CYSTM1*:*LUC*) was lower than that of the shorter 5′ *CYSTM α* sequence (35S:*CYSTM* α:*LUC*), we did not pursue it further.

Previous studies have shown that enhancers effective in dicotyledonous plants often do not exhibit comparable efficacy in monocotyledonous species [3,14]. To evaluate the cross-species potential of our sequence, we performed the wheat germ translation assay. We observed a two-fold increase in LUCIFERASE protein from 35S:*CYSTM α*:*LUC* compared to the control, while the 5′ TMV Ω increased protein production by 2.5-fold (Figure 2C).

### 3.3. 5′ CYSTM α Sequence Enhances Translation in E. coli

**Figure 3:**
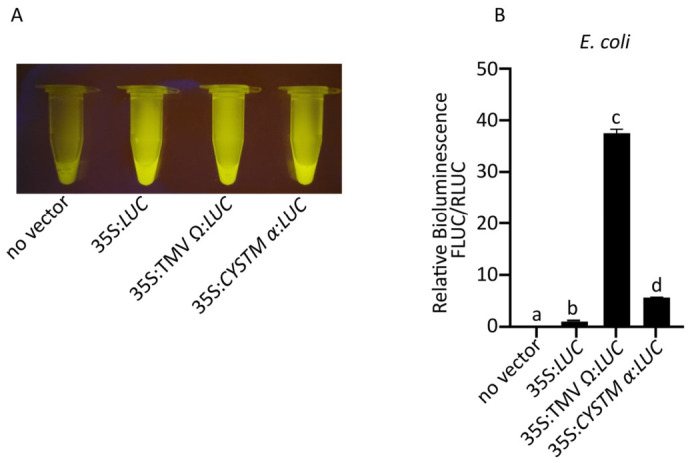
5′ CYSTM α enhances dual-luciferase expression in *E. coli.* (**A**) Qualitative luminescence imaging of *E. coli* transformed with 35S:*LUC*, 35S:TMV Ω:*LUC*, and 35S:*CYSTM α*:*LUC*. Colonies were grown to log phase, illuminated using a blueBox™ S Transilluminator (miniPCR, Cambridge, MA, USA), and imaged with a Samsung Galaxy S20 FE (Thai Nguyen, Vietnam). (**B**) Quantitative Dual-Luciferase^®^ Reporter Assay results showing relative bioluminescence from *E. coli* expressing the 35S:*LUC*(control), 35S:TMV Ω:*LUC*, and 35S*:CYSTM α*:*LUC* constructs. Error bars represent the standard error of the mean. Statistical significance is indicated by letters (a–d) (*p* < 0.01, one-way ANOVA).

Previous studies have shown that translational enhancers of eukaryotic origin typically do not function effectively in prokaryotic systems due to fundamental differences in their translational and regulatory mechanisms [16,17,18]. To evaluate 5′ *CYSTM α* translational enhancer ability in a prokaryotic context, we tested the dual-luciferase construct in *E. coli* (Figure 3A). Dual-luciferase assay results revealed a 5-fold increase in relative bioluminescence for the 35S:*CYSTM α*:*LUC* construct compared to the control, and a 38-fold increase for the 35S:TMV Ω:*LUC* construct relative to the same control (Figure 3B). These findings indicate that while the 5′ *CYSTM α* sequence enhances translation in *E. coli*, its activity is substantially lower than that of TMV Ω.

## 4. Discussion

Translation constitutes a central layer of gene expression control and is essential for coordinating plant development and responses to environmental cues [33]. This process is highly dynamic and mechanistically complex, relying on coordinated interactions among mRNAs, transfer RNAs, and the ribosomal machinery, and is regulated through both cis- and trans-acting factors that integrate endogenous states with external signals [33].

Within this regulatory landscape, 5′ untranslated regions (5′ UTRs) play a particularly important role by encoding sequence and structural features that influence ribosome recruitment and translation initiation efficiency [1,2]. Despite their central function, relatively few endogenous plant 5′ UTRs have been characterised in detail, and most mechanistic insight has come from viral leaders [18,19,34]. Identifying plant-derived UTR elements that confer robust translational control therefore remains an important objective for understanding gene regulation at the post-transcriptional level.

In this study, we identified a short, 79-nucleotide 5′ UTR fragment from the *A. thaliana CYSTM1* gene (5′ *CYSTM* α) that significantly enhances protein translation across multiple platforms, outperforming the widely used TMV Ω enhancer in *A. thaliana* and *N. benthamiana*, and exhibiting robust activity in *E. coli* and wheat germ extract.

The activity of *CYSTM α* should also be considered in the context of established translational enhancers. Synthetic and endogenous leaders such as synJ and ADH-derived 5′ UTRs have previously been reported to enhance reporter output strongly in plant systems, and in some cases, by substantially larger fold-changes than those observed here, although their activity is often tissue-, species-, and construct-dependent [20,21,24]. For example, synJ enhances transgene expression in dicot systems but shows marked context dependence, whereas NtADH can drive strong translation in *N. benthamiana* and *Arabidopsis* suspension cells yet performs poorly in rice [21,24]. In comparison, *CYSTM α* does not represent the highest absolute level of enhancement reported for a plant leader sequence. Rather, its significance lies in its compact size, endogenous *Arabidopsis* origin, and the fact that its effect is supported by unchanged transcript abundance and increased translation efficiency. Thus, *CYSTM α* is best viewed as a promising endogenous translational enhancer that performs strongly in the systems tested here, rather than as a universally superior alternative to all existing enhancer elements.

In both transient and stable assays, 5′ *CYSTM* α conferred significantly higher expression than TMV Ω. Its activity within a polycistronic RUBY reporter further indicates that the enhancer remains effective within complex transcript architectures, supporting a role for 5′ *CYSTM α* as a broadly acting translational regulator.

Mechanistically, the enhancement mediated by 5′ *CYSTM* α appears to be post-transcriptional, which is consistent with previous findings for TMV Ω and ADH-derived UTRs [18,22,24]. While qRT-PCR analysis showed no significant difference in transcript abundance, protein accumulation was substantially increased, suggesting that translation efficiency might be the primary driver of increased protein production.

To assess whether RNA structure contributes to this activity, in silico secondary structure prediction of the 79 nt *CYSTM α* sequence was performed using RNAfold (Figure A3). The predicted minimum free energy (ΔG = −2.70 kcal/mol) indicates that the sequence is only weakly structured and does not form a highly stable inhibitory fold. Instead, *CYSTM α* adopts local stem–loop features while retaining substantial unpaired regions. Such structural properties are consistent with models of translational regulation in which accessible RNA architecture facilitates ribosome recruitment and scanning [35].

**Figure A1:**
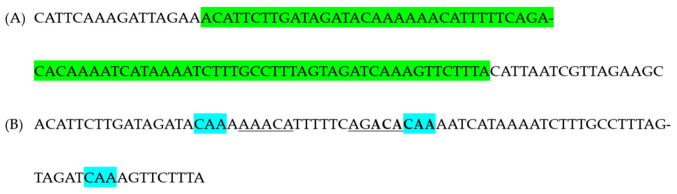
(**A**) Complete 5′ UTR of the *CYSTM1* gene with the 5′ CYSTM α sequence highlighted in green. (**B**) 5′ CYSTM α sequence with CAA motifs highlighted in blue; DRACH motifs are underlined; and the AMAYAA motif is indicated in bold.

**Figure A2:**
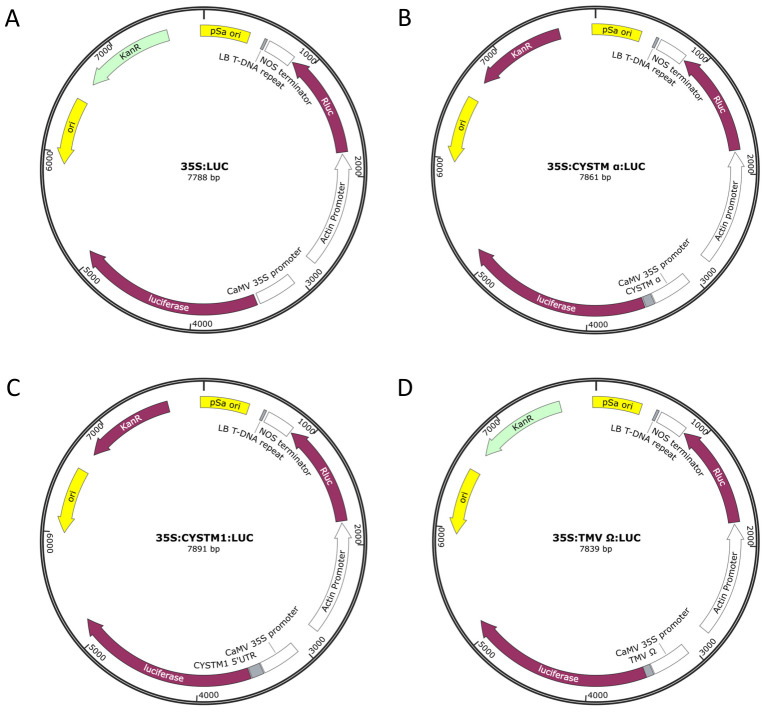
Plasmid maps of (**A**) 35S:*LUC*, (**B**) 35S:TMV Ω*:LUC*, (**C**) 35S:*CYSTM1:LUC*, and (**D**) *35S:CYSTM α:LUC*.

**Figure A3:**
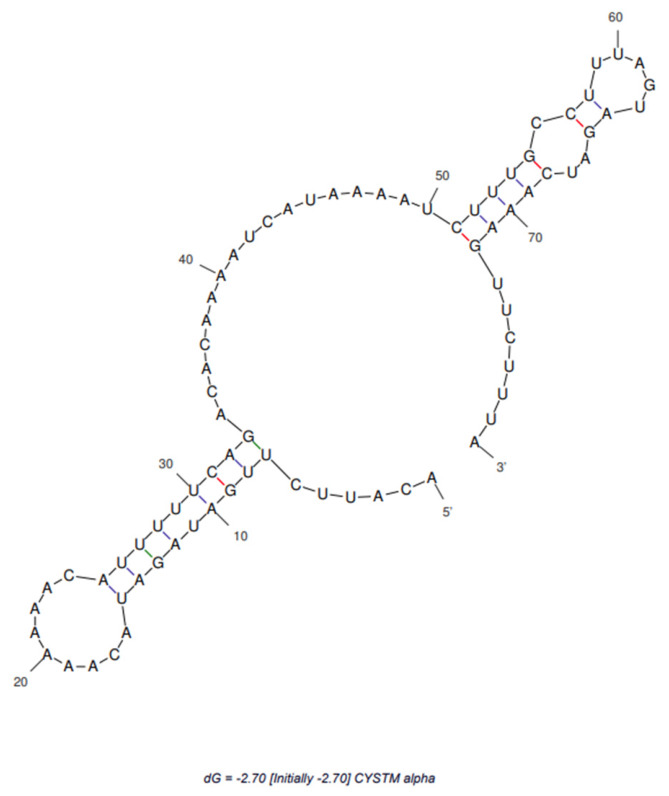
Predicted secondary structure of the CYSTM α 5′ UTR. Secondary structure of the 79-nucleotide *CYSTM α* 5′ untranslated region predicted using RNAfold. The minimum free energy (MFE) structure is shown (ΔG = −2.70 kcal/mol). Nucleotide positions are indicated, and base-pairing interactions are shown by connecting lines. The red lines indicate base-pairing interactions that are highly probable.

A key limitation of the present study is the absence of direct mechanistic validation. While sequence features within *CYSTM α* suggest potential roles in translation regulation, targeted motif mutagenesis and ribosome-level analyses were not performed. In contrast, TMV Ω’s activity is known to depend on poly (CAA) motifs that recruit the HSP101 chaperone and translation initiation factors [18,36,37]. This distinction highlights an important difference between the two elements. Furthermore, the relative performance of *CYSTM α* and TMV Ω appears to be context-dependent. Although *CYSTM α* outperformed TMV Ω in plant-based assays in this study, TMV Ω exhibited stronger activity in *E. coli*, indicating that its functionality is more consistent across divergent systems. This suggests that TMV Ω may rely on more robust or broadly compatible features of translation initiation, whereas *CYSTM α* may depend on context-specific interactions or RNA properties. Therefore, while *CYSTM α* represents a promising endogenous enhancer, TMV Ω remains advantageous in applications requiring predictable performance and established mechanistic understanding. The 5′ *CYSTM* α sequence similarly contains three CAA repeats, two DRACH consensus motifs, and an AMAYAA motif, which have been implicated in enhanced translation initiation via eIF3 binding in other systems (Figure A1) [38,39,40,41,42,43]. However, these observations are correlative, and the present study does not include functional validation of these elements. As such, these features should be interpreted as candidate motifs rather than confirmed determinants of activity. Interestingly, in publicly available m^6^A-SAC-seq data, one of the two m^6^A motifs was methylated, implicating enhanced translation initiation via eIF3 binding [44]. Furthermore, publicly available ribosome profiling data of *Arabidopsis* [45] revealed that *CYSTM1* had 1132 ribosome footprint reads across the 216-codon ORF, corresponding to 758 P-sites, of which approximately 90% were in-frame. Comparison with RNA-seq coverage indicated a translation efficiency of approximately 3.25, which is consistent with highly efficient translation. The high density of ribosome footprints along the coding sequence and the predominant in-frame P-site distribution collectively suggest that the entire ORF is actively translated. Together, these observations suggest that the regulatory properties of 5′ *CYSTM α* reflect evolutionarily encoded features of the *CYSTM1* leader.

A particularly intriguing aspect of 5′ *CYSTM* α is its cross-kingdom activity. While plant-derived enhancers typically fail in prokaryotes due to fundamental differences in translation initiation [46,47,48], *CYSTM α* exhibited an approximately five-fold increase in protein production. However, this effect was consistently lower than that observed for TMV Ω under the same conditions, indicating that its efficiency is context dependent.

This residual activity suggests an overlap with bacterial translation signals, possibly through stable RNA secondary structures or Shine–Dalgarno-like motifs [49,50]. This observation highlights the possibility that certain UTR-encoded features operate through conserved biophysical principles rather than kingdom-specific pathways.

From a genetics perspective, 5′ *CYSTM* α complements existing strategies to enhance gene expression, such as promoter engineering [51], intron-mediated enhancement [11,52], and codon optimisation [12]. Its small size and robust activity make it a valuable tool for dissecting how UTR-encoded motifs integrate with other regulatory layers to shape protein abundance.

Future work should aim to identify the minimal active region, assess structure–function relationships, and test for the presence of m^6^A modifications and protein binding. Ribosome profiling and cross-species testing will further refine our understanding of its mechanism and utility. In summary, 5′ *CYSTM* α is a post-transcriptional enhancer that improves protein yield across diverse hosts. Its cross-kingdom functionality and compatibility with multigene constructs underscore its potential for use in plant and microbial synthetic biology.

## Data Availability

All plasmids generated in this study were deposited in Addgene under the following accession numbers: 247490, 247491, 247492, 247493, and 247568.

## Appendix A

**Table A1:** Effect of different DNA fragments on luciferase bioluminescence.

Tair ID	Gene Name	Transcript Position	Sequence	Effect on Bioluminescence
AT1G05340	CYSTM 1 cysteine-rich TM module stress tolerance protein	5′ UTR	acattcttgatagatacaaaaaacatttttcagacacaaaatcataa aatctttgcctttagtagatcaaagttcttta	Increased
AT1G05340	CYSTM 1 cysteine-rich TM module stress tolerance protein	Exon 1	atgagccagtacgat cacaaccagtctgcag	No effect
AT1G05340	CYSTM 1 cysteine-rich TM module stress tolerance protein	Exon 3	tcttgcggccatgtgttgctgttgtgccctggacatttgcttctaa	No effect
AT3G01420	DOX 1 Peroxidase superfamily protein	Exon 9	gggacttatggcagagaagaaaatcaaaggttttgctatcagtgaga ctgctttttacattttcctcatcatggccaCaag	No effect
AT4G20260	PCAP1 plasma membrane-associated cation-binding protein 1	5′ UTR	gtttggagaagaagaagaacagatcaaatacgaggagagatctcta aagagatttatcgtttcaagtaagtctctttatcaa	No effect
AT4G20260	PCAP1 plasma membrane-associated cation-binding protein 1	Exon 5	agatggtatgattacttctcttgtatgaaaacatctttgtacgtaacaa aaaaatgaaaggaagaaacggaaaggaaca	No effect
AT5G54940	Translation initiation factor SUI1 family protein	Exon 3	atggttgagctagacattcagattccatcagcatacgatcca tttgcagaagctaaagattcagatgcaccaggagctaaaga gaacattcacattcgaatccagcagaggaatgggaaa	No effect
AT1G78380	GSTU19 glutathione S-transferase TAU 19	Exon 2	cgtggctaagtccttgcctgatcctgagaaggttactgaattcgt ctctgagctcaggaagaaatttgtacctgagtaa	No effect
AT1G78380	GSTU19 glutathione S-transferase TAU 19	3′ UTR	tctcttgtcatatggttcggacttaagatgctctctgttttactttc gtgtgttgtctttcttaaataatgcagagcatctgtgaggtggtt tgagtgtagagcatc	No effect
AT3G22260	Cysteine proteinases superfamily protein	Exon 1	aaatcaaacataacaaaaaccaacaaaacaaactcaaaaggattcttcttcaaacacaacatcttca agaaaacat	No effect
AT1G13320	PP2AA3 protein phosphatase 2A subunit A3	Exon 2	tgtctatggttgatgagcctttatacccgattgctgtgcttatcgacgagctaaaaaacgatgatattca gcgtagattgaac	No effect
